# Supplementary material for: Stabilizing Halogen-Bonded Complex between Metallic Anion and Iodide
Source: Molecules. 2022 Nov 21;27(22):8069. doi: 10.3390/molecules27228069 (PMC9692347; doi:10.3390/molecules27228069)
Supplement: Supplementary file 1 [file molecules-27-08069-s001.zip › molecules-2030007-supplementary.pdf]

Supporting Information

Stabilizing Halogen-Bonded Complex between Metallic Anion and Iodide

Fei Ying[1], *Xu Yuan*[2,3], *Xinxing Zhang*[2,3]*, *Jing Xie*[1]*

1 Key Laboratory of Cluster Science of Ministry of Education, Beijing Key Laboratory of Photoelectronic/Electrophotonic Conversion Materials, School of Chemistry and Chemical Engineering, Beijing Institute of Technology, Beijing 100081, China

2 College of Chemistry, Key Laboratory of Advanced Energy Materials Chemistry (Ministry of Education), Renewable Energy Conversion and Storage Center (ReCAST), Tianjin Key Laboratory of Biosensing and Molecular Recognition, Shenzhen Research Institute, Frontiers Science Center for New Organic Matter, Nankai University, Tianjin, 300071, China.

3 Haihe Laboratory of Sustainable Chemical Transformations, Tianjin 300192, China.

Corresponding Authors

Xinxing Zhang, e-mail: zhangxx@nankai.edu.cn

Jing Xie, e-mail: jingxie@bit.edu.cn

Table of Contents

Table of Contents		S2
Figure S1	Optimized structure of $Mn(CO)_5^-$ anion	S3
Figure S2	CID fragments of the $Mn(CO)_5^-$ anion	S4
Figure S3	Optimized structures of $CH_3I{-}Mn(CO)_3^-$	S5
Figure S4	Additional conformers of RC	S6
Cartesian coordinates of calculated structures		S7

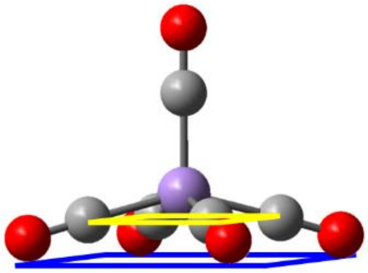

Figure S1. Optimized structure of $Mn(CO)_5^-$ anion. Color code: C, grey; O, red; Mn, purple.

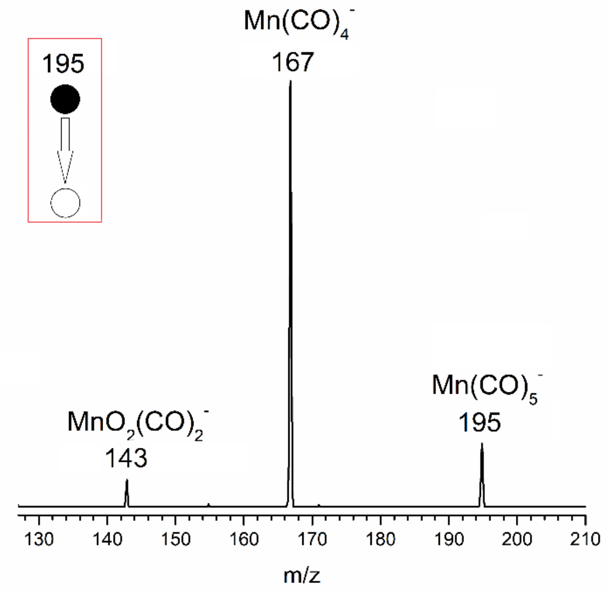

Figure S2. CID fragments of the $Mn(CO)_5^-$ anion.

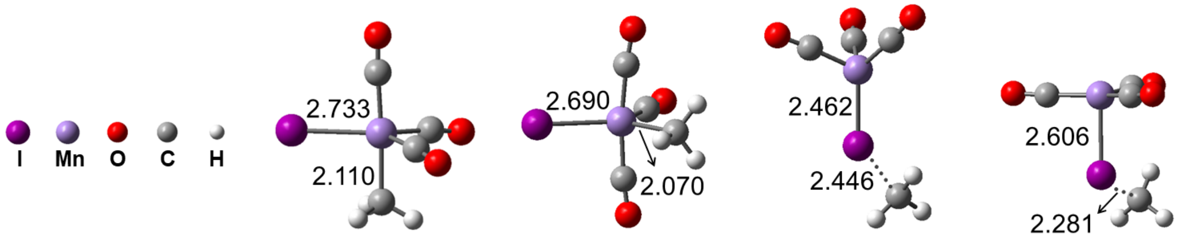

relative energetics (kcal/mol)				
$\Delta(E_{elec}+ZPE)$	0.0	4.7	54.5	77.0
ΔE_{elec}	0.0	4.8	55.7	78.0
$\Delta H_{298.15K}$	0.0	4.8	55.1	77.6
$\Delta G_{298.15K}$	0.0	4.5	52.8	75.7

Figure S3. Optimized structures of $CH_3I{-}Mn(CO)_3^-$. Relative energetic values are reported.

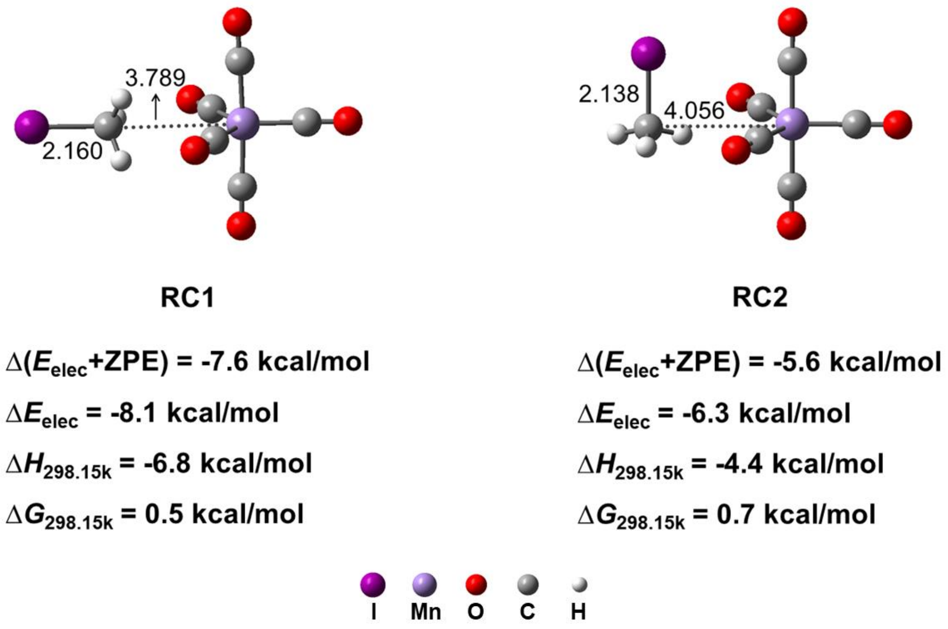

Figure S4. Additional conformers of the pre-reaction complex (RC) of CH_3I reacting with $Mn(CO)_5^-$. Energetic values are given in relative to the total energy of isolated $Mn(CO)_5^-$ and CH_3I.

Cartesian coordinates (Å) of all calculated structures for $Mn(CO)_5^-$ + CR_3I reactions (R = H, F).

$Mn(CO)_5^-$

Mn	0.00000000	0.00000000	-0.01271400
C	0.00000000	1.77338200	0.43836200
C	-1.77338200	0.00000000	0.43836200
C	0.00000000	-1.77338200	0.43836200
C	1.77338200	0.00000000	0.43836200
C	0.00000000	0.00000000	-1.78591700
O	-2.88192900	0.00000000	0.75057700
O	0.00000000	2.88192900	0.75057700
O	0.00000000	0.00000000	-2.93822600
O	2.88192900	0.00000000	0.75057700
O	0.00000000	-2.88192900	0.75057700

XC

Mn	1.57003600	-0.00048900	0.00044000
C	1.14385200	1.75225500	0.34711600
C	1.14040700	0.34712300	-1.75117200
C	1.13917500	-1.75152200	-0.34951800
C	1.13973500	-0.34957600	1.75166200
C	3.34562600	-0.00106800	0.00123200
O	0.84083400	0.56245000	-2.83940800
O	0.84696400	2.84134900	0.56155400
O	4.49626500	-0.00117000	0.00159700
O	0.84029400	-0.56714800	2.83946100
O	0.83803500	-2.83870700	-0.56775200
I	-2.08191700	0.00075000	0.00043600
C	-4.24928500	0.00161800	0.00069200
H	-4.58802200	1.01354100	0.19309000
H	-4.58874400	-0.33756000	-0.97166500
H	-4.58869900	-0.67067900	0.78079500

XC'

Mn	-1.99192500	0.00006700	0.00007400
C	-1.62118400	-0.17552800	1.80610800
C	-1.62483200	-1.80685400	-0.17552200
C	-1.62295200	0.17552200	-1.80628900
C	-1.62493300	1.80701600	0.17561900
C	-3.77608300	0.00001300	0.00084200
O	-1.36761500	-2.91597400	-0.28373500
O	-1.36079000	-0.28362700	2.91453500

O	-4.92235400	-0.00046600	0.00131400
O	-1.36656400	2.91591800	0.28332200
O	-1.36366500	0.28371600	-2.91498000
I	1.23201700	0.00025100	-0.00011200
C	3.48684800	-0.00024900	-0.00012700
F	4.00910800	-0.12826600	1.23598100
F	4.00895900	-1.00699200	-0.72883800
F	4.00948300	1.13403200	-0.50751700

RC

Mn	2.22667400	0.03446600	-0.00038600
C	1.45198200	-0.22056400	1.60336500
C	1.58500400	1.76073100	0.00027400
C	1.40019400	-0.23572700	-1.57631100
C	2.75238000	-1.73532100	-0.00044700
C	3.93313900	0.59802800	-0.03012600
O	1.15551300	2.82361500	-0.00103100
O	0.92699000	-0.38480200	2.62004100
O	5.02610500	0.96192700	-0.04885700
O	3.06655800	-2.83644900	-0.00009600
O	0.84269000	-0.41095800	-2.57334700
C	-1.56579100	-0.06424300	0.00606300
H	-1.24116400	0.93231300	-0.26223200
H	-1.25124200	-0.79325700	-0.72775300
H	-1.25424500	-0.33266800	1.00609200
I	-3.72461300	-0.04739900	0.00005500

RC1

Mn	-2.22195200	0.00308600	-0.00195700
C	-1.44042200	-0.01381300	1.61801000
C	-2.20219800	-1.84165900	-0.01027400
C	-1.32352800	-0.01210200	-1.56198400
C	-2.13127100	1.84544700	-0.00658200
C	-4.01769100	0.03696400	-0.05853900
O	-2.17594200	-2.98673600	-0.01895500
O	-0.91317300	-0.02536800	2.64677600
O	-5.16901400	0.05879000	-0.09513100
O	-2.05972300	2.98866000	-0.01307400
O	-0.71821700	-0.02205700	-2.54632400
C	1.56585300	-0.02438900	0.06599000
H	1.23829600	-0.88934100	-0.49408100
H	1.22095800	0.89170100	-0.39357700

H	1.28501800	-0.08439800	1.10848000
I	3.72431400	-0.00083200	-0.00449000

RC2

Mn	1.75056800	-0.08417000	-0.00153100
C	0.92108400	-1.73710000	-0.04762200
C	1.04539200	0.22382200	1.62244200
C	2.56378700	1.56751300	0.04344300
C	0.84656100	0.40464900	-1.47286700
C	3.35913500	-0.88382400	-0.14671100
O	0.56917300	0.44462100	2.65154800
O	0.41552000	-2.76290300	-0.07768700
O	4.39021800	-1.39195800	-0.24074700
O	0.23723800	0.74171600	-2.39610100
O	3.06451000	2.59846900	0.07168200
C	-1.86783000	1.74597300	-0.07446600
H	-2.44914600	2.42292000	0.53875000
H	-1.85370400	2.04154700	-1.11580800
H	-0.86124800	1.59659600	0.30050900
I	-2.81551200	-0.16835100	0.01320600

RC'

Mn	-2.53040500	0.04105100	-0.00001300
C	-1.75061600	-0.41040400	1.55544400
C	-3.43749800	-1.56322900	-0.00035200
C	-1.75137800	-0.40822700	-1.55640400
C	-1.62655200	1.64690900	0.00138900
C	-4.09820000	0.92481000	0.00021700
O	-4.00252800	-2.56037400	-0.00034100
O	-1.24438600	-0.71065400	2.54922200
O	-5.10335900	1.49236200	0.00043800
O	-1.06113900	2.64434800	0.00244300
O	-1.24558100	-0.70705300	-2.55081000
C	1.59252600	-0.01306500	-0.00017700
F	1.13296900	-1.24450800	-0.00043300
F	1.17468300	0.61948500	-1.07521800
F	1.17456900	0.61909200	1.07505600
I	3.76615900	-0.06231500	-0.00005000

TS1

Mn	-1.97858700	0.00000300	-0.00513600
C	-1.69605100	0.00186400	1.82449000
C	-1.61654300	-1.81677400	0.01300600

C	-1.52616000	-0.00188800	-1.80242900
C	-1.61611200	1.81673400	0.00926900
C	-3.76400000	0.00011400	-0.08228800
O	-1.34795300	-2.92826700	0.02324500
O	-1.47689800	0.00302100	2.94700300
O	-4.90777900	0.00018700	-0.13332700
O	-1.34724400	2.92817800	0.01722700
O	-1.19814400	-0.00301700	-2.89784600
C	0.89224300	-0.00000200	0.00202300
H	0.85808700	-0.92893300	-0.53265800
H	0.85813900	0.92683500	-0.53628500
H	0.84762300	0.00209600	1.07335300
I	3.49216400	-0.00002200	0.01300300

TS1'

Mn	2.27901400	0.00026400	-0.00712300
C	1.89472200	1.80426400	-0.10762000
C	1.73372900	-0.13587400	-1.76588800
C	1.91966600	-1.80360100	0.16196300
C	2.04938800	0.13500800	1.81936000
C	4.06677000	0.00125900	-0.15427900
O	1.33720600	-0.21908400	-2.83613600
O	1.60232300	2.90796600	-0.16996300
O	5.20666300	0.00184800	-0.25320300
O	1.84299700	0.21768700	2.94059700
O	1.64151000	-2.90793600	0.26403200
C	-0.81260200	-0.00114300	-0.01163000
F	-0.81673100	-1.16717400	-0.53611700
F	-0.79788000	0.13032700	1.25956500
F	-0.82010600	1.03314800	-0.76314600
I	-3.64563300	0.00044100	0.02493100

TS2

Mn	1.51253500	-0.11239600	-0.06103700
C	0.57522500	-1.69030500	0.37730700
C	1.75760200	0.15630300	1.75682300
C	1.92914600	1.68066200	-0.27956600
C	0.57528900	0.03737700	-1.68038600
C	3.02842900	-0.91930900	-0.55602100
O	1.87650500	0.36958900	2.87527400
O	0.06335000	-2.65702800	0.67873800
O	4.01796300	-1.40187700	-0.86977900

O	0.01703300	0.18106500	-2.66238000
O	2.03640100	2.81948700	-0.34286800
C	-0.83258700	2.01387800	0.53702200
H	-1.81797200	2.26030200	0.88439600
H	-0.58347000	2.35130000	-0.45626700
H	-0.05661800	2.06788000	1.28078800
I	-2.67252800	-0.11379500	0.02743500

TS2'

Mn	-1.70940000	-0.27342400	0.00012000
C	-0.56399900	-0.93888300	-1.36536800
C	-2.49165600	0.81936900	-1.30686200
C	-2.49121200	0.82142200	1.30566400
C	-0.56373400	-0.93705600	1.36629000
C	-2.94608100	-1.56131800	0.00120500
O	-2.98084100	1.47843800	-2.09665000
O	0.06505900	-1.29593300	-2.23875600
O	-3.73663300	-2.38675200	0.00191000
O	0.06551900	-1.29293100	2.24001100
O	-2.98017500	1.48176500	2.09452700
C	0.99719200	1.53650100	-0.00058100
I	2.70424100	-0.58791900	0.00008000
F	0.31203100	1.92275100	1.09624700
F	2.07742400	2.34137700	-0.00129600
F	0.31095800	1.92236200	-1.09691100

PC1

Mn	1.37321600	-0.00012600	0.00297200
C	2.60597700	-1.31111400	0.48656000
C	0.30671600	-1.37753500	-0.87976600
C	0.30942400	1.38375200	-0.87290100
C	2.60960400	1.30558800	0.49129100
C	0.41962400	-0.00269100	1.64853000
O	-0.12901600	-2.21718500	-1.49111400
O	3.41317300	-2.08614200	0.68468700
O	-0.01687500	-0.00380800	2.69013600
O	3.41945700	2.07733000	0.69148700
O	-0.12561400	2.22690700	-1.47986800
C	2.44885900	0.00448100	-1.90166600
H	1.74072400	-0.00192400	-2.73239500
H	3.07868400	0.88777800	-2.02578100
H	3.09188200	-0.86960500	-2.02280700

I	-2.77230000	-0.00009100	0.07758100
**PC1'**			
Mn	0.93951700	0.38451400	-0.00063800
C	1.96691500	1.22400800	1.33763500
C	0.00253000	-0.65919800	1.40665000
C	0.00398100	-0.66721400	-1.40247600
C	1.96898000	1.21572800	-1.34225500
C	-0.36381100	1.82517000	-0.00533500
O	-0.37183400	-1.27348200	2.26830500
O	2.61460200	1.70055400	2.13386400
O	-0.94799300	2.78902600	-0.00851900
O	2.61846800	1.68670600	-2.14034800
O	-0.37026500	-1.28663900	-2.26050400
C	2.44300100	-1.09024600	0.00275200
F	2.44559900	-1.94520400	1.07516700
F	2.45958000	-1.93387500	-1.07872500
F	3.72495800	-0.57707400	0.01355900
I	-3.12514300	-0.17974100	0.00003300
**TS3**			
Mn	-1.35623300	-0.06512000	0.04624100
C	-1.97686800	1.61106500	-0.40080000
C	-0.20868200	0.78022100	1.40423500
C	-0.21965800	-1.64900400	-0.09610400
C	-2.53888600	-0.75851700	-1.23077400
C	-2.43223300	-0.68251200	1.37763600
O	0.27754600	1.31021500	2.26941500
O	-2.53437700	2.62775500	-0.55252200
O	-3.16276000	-0.99450100	2.19567800
O	-3.29266400	-1.15000800	-1.98308200
O	0.25601000	-2.66778300	-0.18039300
C	-0.51412200	1.38418500	-1.53153100
H	0.00076600	2.29511900	-1.24148500
H	0.24953300	0.61102900	-1.61573300
H	-1.02704200	1.51821600	-2.48011300
I	2.82406100	0.00161400	-0.13108400
**TS3'**			
Mn	-1.08516600	-0.61386400	0.04485900
C	-1.97055200	0.77301700	0.96997800
C	0.17110800	-0.69953100	1.59311500
C	0.15548200	-1.53203900	-1.19871700

C	-2.40995700	-0.50648000	-1.30015700
C	-1.87699700	-2.13431900	0.67142100
O	0.63344300	-0.83375100	2.61026000
O	-2.62022900	1.40820200	1.70504500
O	-2.42054800	-3.03916300	1.09480300
O	-3.25242900	-0.48102200	-2.05529300
O	0.66947900	-2.16266900	-1.97724200
C	-1.03941300	1.97440600	-0.15411600
F	-0.34483800	2.79532800	0.63597300
F	-0.25205800	1.68555900	-1.21795400
F	-2.06328900	2.69979100	-0.66469400
I	2.80783100	0.08183800	-0.08322900
**PC2**			
Mn	-0.72000100	0.35574700	-0.00001500
C	-0.67608000	0.05406100	1.85394900
C	-2.51381600	0.77366900	-0.00002200
C	-0.67609400	0.05425300	-1.85398700
C	-0.19844500	2.15584300	0.00006700
O	-0.66499700	-0.22373600	2.95603600
O	0.09126900	3.25519800	0.00012900
O	-0.66502600	-0.22341100	-2.95611200
O	-3.61622500	1.04987000	-0.00005100
C	-1.31610000	-1.68152100	-0.00004300
O	-2.49373800	-1.99823200	0.00042200
C	-0.28906500	-2.80478500	-0.00047000
H	-0.79411600	-3.77210600	-0.00063000
H	0.36382200	-2.70504100	0.86818300
H	0.36366300	-2.70462900	-0.86918800
I	2.09196200	-0.11129300	0.00003100
**PC2'**			
Mn	-0.25966900	0.99272800	0.00001500
C	-0.12334500	0.79455500	-1.88169400
C	0.30359900	2.75153800	0.00009000
C	-0.12314300	0.79435300	1.88168500
C	-2.07346300	1.49466500	0.00015400
O	0.00997500	0.64700400	-2.99693300
O	-3.15884800	1.82687400	0.00023500
O	0.01027600	0.64670300	2.99689900
O	0.61467800	3.84364800	0.00015100
C	1.79303200	0.50189900	-0.00020700

O	2.68309100	1.32577800	-0.00064000
C	2.32311400	-0.97235100	0.00013900
F	3.66209300	-1.05656400	-0.00011200
F	1.89797900	-1.63337100	-1.08493300
F	1.89844200	-1.63273400	1.08578000
I	-1.40579300	-1.59287200	-0.00010800

$Mn(CO)_4I^-$

Mn	-0.74464500	-0.00012200	-0.00009000
C	-0.78121400	1.91151600	0.00016800
C	-1.91372600	0.00059200	-1.52011600
C	-1.91166500	-0.00087200	1.52191800
C	-0.78057900	-1.91179500	-0.00118400
O	-0.87446100	3.04780200	0.00014400
O	-0.87338900	-3.04811200	-0.00186100
O	-2.64071600	-0.00141200	2.40948300
O	-2.64398700	0.00123800	-2.40674000
I	2.02263500	0.00019400	-0.00020100

$CH_3I-Mn(CO)_3^-$

Mn	0.76115600	-0.02265000	-0.00039900
C	2.04281400	-0.12234100	-1.26699400
C	2.04095400	-0.12287000	1.26787200
C	0.58970000	1.83067500	-0.00001800
O	2.84272300	-0.30513200	2.06446000
O	2.84578300	-0.30417200	-2.06245400
O	0.54742800	2.96966800	0.00047700
C	0.83725900	-2.13091300	-0.00062900
H	1.82159800	-2.60958300	-0.00053100
H	0.28790400	-2.48379400	0.87726400
H	0.28802000	-2.48370000	-0.87862600
I	-1.96940100	-0.14088500	-0.00017700

$CF_3I-Mn(CO)_3^-$

Mn	0.48445500	0.50213200	0.00002700
C	1.72046200	0.83677800	-1.30780400
C	1.72163900	0.83578400	1.30690500
C	-0.18826400	2.26177200	0.00083600
O	2.48625800	1.00172000	2.13421600
O	2.48427600	1.00348000	-2.13569300
O	-0.56063500	3.33477500	0.00142700
C	1.14412800	-1.47440100	-0.00012000
I	-2.07891900	-0.28907000	-0.00003000

F	0.76251200	-2.22403000	1.08138100
F	2.52584000	-1.62813400	-0.00373300
F	0.75657900	-2.22694500	-1.07738200
